# Chemokines in Primary Liver Cancer

**DOI:** 10.3390/ijms23168846

**Published:** 2022-08-09

**Authors:** Monika Zajkowska, Barbara Mroczko

**Affiliations:** 1 Department of Neurodegeneration Diagnostics, Medical University of Bialystok, 15-269 Bialystok, Poland; 2 Department of Biochemical Diagnostics, Medical University of Bialystok, 15-269 Bialystok, Poland

**Keywords:** liver cancer, cytokines, pathogenesis, liver disease, chemokines

## Abstract

The liver is responsible for extremely important functions in the human body. In the liver’s structure, we distinguish between connective tissue (stroma) and parenchyma, the latter of which is formed from the basic structural and functional units of the liver—hepatocytes. There are many factors, that negatively affect the liver cells, contributing to their damage. This may lead to fibrosis, liver failure and, in consequence, primary liver cancer, which is the sixth most commonly diagnosed malignancy and the fourth leading cause of cancer death worldwide. Chemokines are a large family of secreted proteins. Their main role is to direct the recruitment and migration of cells to sites of inflammation or injury. Some authors suggest that these proteins might play a potential role in the development of many malignancies, including primary liver cancer. The aim of this study was to evaluate and summarize the knowledge regarding liver diseases, especially primary liver cancer (HCC) and the participation of chemokines in the development of this malignancy. Chemokines involved in the initiation of this type of tumor belong mainly to the CC and CXC chemokines. Their significant role in the course of hepatocellular carcinoma proves their usefulness in detecting and monitoring the course and treatment in patients with this disease.

## 1. Introduction

The liver is responsible for extremely important functions in the human body. Among them we can mention: nutrients metabolism, storage of vitamins, protein synthesis, bile secretion and detoxication. Serious damage of liver cells leads to dysfunction of this organ, which may cause secondary dysfunction of the whole organism. The most frequently observed symptoms of liver and bile duct diseases include fatigue, apathy, excessive drowsiness, impaired concentration, dyspepsia, bloating, nausea, vomiting, anorexia and pain in the right side of upper abdomen [1]. Factors that are connected with hepatocyte damage include: alcohol, viruses, drugs, toxins, heavy metals or metabolic products. Moreover, obesity has been shown to be an independent risk factor for fibrosis and other chronic liver diseases. Over the last 20 years, the number of overweight and obese people has increased alarmingly. In addition, an increased risk of developing type 2 diabetes, hypertension and lipid disorders also have an adverse effect on the liver [2]. Fibrosis and chronic liver disease may lead to a more serious disorder—primary liver cancer. The most common primary liver cancers include HCC (hepatocellular carcinoma) and CCC (cholangiocarcinoma). The most important risk factor of primary liver cancer is liver cirrhosis, regardless of its cause [3]. A summary of the most important risk factors is presented in Table 1.

At present, the mortality from liver cancer is very high due to low and late detection of these tumors. The existing methods in screening diagnosis are based solely on imaging diagnostics and biopsy. Less invasive methods such as the concentration of tumor markers are not popular due to their low diagnostic sensitivity and specificity [4].

Therefore, new markers are still being sought to diagnose the early stage of the disease. Some authors suggest that chemokines might be a potential biomarker of liver cancer. These low-mass proteins belong to the cytokine group and are involved in the pathogenesis of cancer by participating in the inflammatory processes [5].

Chemokines may increase the migration of specific cells to inflamed tissues, and thus participate in the pathogenesis of certain diseases. The complex role of chemokines is related both to their role in modulating the inflammatory response as well as in initiating and sustaining processes related to tumor formation. These proteins are believed to take part in the regulation of growth, cell proliferation and apoptosis, as well as in angiogenesis and modification of the anti-tumor response (including its inhibition), and the formation of tumor metastasis. Therefore, it is very important to evaluate and summarize knowledge regarding liver diseases, especially primary liver cancer (HCC) and the participation of chemokines in the development of this malignancy, which was the aim of this study.

## 2. Liver Diseases

Common liver diseases include non-alcoholic fatty liver disease (steatosis), alcoholic liver disease, drug-induced liver injury and hepatitis. Each of these diseases is a factor that predisposes to the development of primary liver cancer. 

### 2.1. Non-alcoholic Fatty Liver Disease

Non-alcoholic fatty liver disease (NAFLD) is currently the most common chronic liver disease. It is characterized by excess retention of triglycerides and affects about 25–45% of the population. It is directly related to diseases such as obesity and diabetes [6]. It has been proven that a sedentary lifestyle significantly affects the incidence of NAFLD, independent of body mass index (BMI) [7]. NAFLD is recognized as one of the HCC etiology factors, even without accompanying cirrhosis; it occurs in the absence of high alcohol consumption and can lead to NASH (non-alcoholic steatohepatitis), fibrosis, cirrhosis and HCC [8,9]. In addition, NAFLD and NASH significantly increase the risk of cardiovascular disease [9]. The pathogenesis of NAFLD is based on a cascade of events (insulin resistance, abnormal hepatocyte lipid accumulation, oxidative stress, inflammation, apoptosis, fibrosis) leading to the development of NASH, but the precise mechanism of the development and progression of NAFLD still remains unclear, although involvement of many factors, such as oxidative stress, fatty acid synthesis and inflammation, have been identified [10,11,12,13]. It is believed that the thyroid hormones can also play a significant role in the pathogenesis of NAFLD. This is connected with their role in lipid metabolism, as hypothyroidism may cause hypercholesterolemia [14,15,16]. Liver biopsy is known as the gold standard of NAFLD diagnosis. It is graded as four types according to histological examination. Type 1 is connected only with fatty liver, type 2 with fat accumulation and lobular inflammation, type 3 with additional ballooning degeneration and type 4 with additional Mallory hyaline or fibrosis [17,18,19]. Kanwal et al. [20] reported that 490 patients, from a total of 296,707 with NAFLD, developed HCC.

### 2.2. Alcoholic Liver Disease

Alcoholic liver disease (ALD) is a leading cause of cirrhosis and primary liver cancer [21]. An alcoholic fatty liver (steatosis) is an initial stage of ALD. It can develop into alcoholic steatohepatitis (hepatic inflammation with injury) or alcoholic hepatitis, which is a more severe form with a very poor prognosis [22]. Some patients with chronic liver injury may progress into hepatic fibrosis and cirrhosis in the future, developing HCC (hepatocellular carcinoma) [23,24,25]. The pathogenesis of ALD can be mainly divided into liver injury connected with ethanol consumption (as the main cause), immunological response to injury with an inflammatory process and intestinal permeability with microbiome changes [26].

Although the consumption of alcohol in European countries is decreasing, it still remains at a very high level. The pathophysiology of ALD is still incompletely understood, but relates largely to the direct toxic effects of alcohol [27]. According to the World Health Organization (WHO), 60–80% of liver-related mortality in Europe is due to excessive alcohol consumption [28]. To distinguish an alcoholic from a non-alcoholic basis is difficult. In the diagnosis, ANI (ALD/NAFLD index) can be useful, which uses the MCV value (mean corpuscular volume of erythrocytes), AST/ALT ratio, BMI and gender to identify ALD patients [29,30]. Abstinence is considered to be the most effective therapy for ALD. Drinking cessation terminates alcoholic steatosis and significantly improves survival in cirrhotic patients [31].

### 2.3. Drug-Induced Liver Injury

Drug-induced liver injury (DILI) is considered to be the most important cause of acute liver failure in the USA and Europe, even though it represents less than 1% of all cases of liver disease detected by gastroenterologists [32]. DILI can be classified as intrinsic or idiosyncratic. Intrinsic DILI is predictable on the basis of dose and the pharmacological properties of overdosed drugs (i.e., APAP—acetaminophen). On the other hand, idiosyncratic DILI is unpredictable by the same features. It is believed that the second type of DILI can be a consequence of interactions between environmental conditions, properties of the drug and host factors. DILI is a very complicated medical condition as it can imitate almost any acute or chronic hepatobiliary condition. Additionally, there are currently no specific diagnostic biomarkers or tests available for the differentiation of this condition. Therefore, DILI is monitored and diagnosed nowadays on the basis of commonly used general liver injury serum biomarkers (alanine aminotransferase—ALT, aspartate aminotransferase—AST, alkaline phosphatase—ALP, and total bilirubin—TBL) [33,34]. There are studies showing the usefulness of other parameters, such as glutamate dehydrogenase—GLDH, malate dehydrogenase—MDH, high-mobility group box 1 protein—HMGB1, keratin 18—K18, and microRNA—miR-122 and miR-129 in human and animal models, mainly based on acetaminophen overdose [35,36,37,38,39,40,41,42,43]. The research of Onji et al. [44] has shown that, in older people, DILI requires significantly longer hospitalization than in younger people. It may be connected with increased susceptibility to adverse drug reactions in patients aged >65 years old [2].

### 2.4. Hepatitis

Hepatitis is a group of infectious inflammatory diseases of the liver that is characterized by liver damage caused by hepatotropic viruses (hepatitis viruses). There are several types of viruses that cause different forms and symptoms of hepatitis. The most common are hepatitis virus A (HAV), B (HBV), C (HCV) and E (HEV) [45,46,47,48,49]. Among them, hepatitis B, which is the most common infectious disease in the world, and hepatitis C known as ‘the silent killer’, which has no symptoms in the early stages of infection and usually appears after several years, deserve special attention. These two types of viral hepatitis constitute the main risk factors for developing hepatocellular carcinoma preceded by liver failure and cirrhosis [50,51].

HBV is the most common HCC etiological agent in the world. The areas particularly vulnerable to HBV infection include Asian, African, Middle Eastern and Eastern/Central European countries. Risk factors for HCC associated with HBV infection include, but are not limited to, persistent high viral replication, older age (>40 years) with HBeAg seroconversion, male gender, mother-to-child transmission and other carcinogens such as smoking and alcohol. Liver cancer prophylaxis includes primary prevention with general-population-targeted vaccination against HBV, secondary prevention with an antiviral agent in high-risk patients with chronic HBV infection and tertiary prevention with an antiviral agent to prevent relapse in patients successfully treated for cancer liver. Unfortunately, despite the high prophylactic efficacy in the prevention of liver cancer, the number of new cases is still increasing [52].

HCV is also one of the most important risk factors for primary cancer. It is a non-cytopathic virus that damages the liver through immune mechanisms. The virus plays a significant role in the development of HCC. HCV genotype 3 is also associated with a higher risk of HCC than other genotypes [53]. The mechanism by which HCV genotype 3 can be involved in hepatocarcinogenesis is not fully understood, but it may be related to the downregulation of the phosphatase and tensin (PTEN) homolog gene, which acts as a tumor suppressor [54,55]. Currently, the identification of infected patients is a major challenge to reduce HCV morbidity and mortality. The asymptomatic progression of the early stages of the disease makes this challenge even more difficult to overcome. Consequently, efforts should be made to raise the awareness of society and health care professionals about HCV underdiagnosis [56]. It is also extremely important to shorten the path from diagnosis to highly effective HCV treatment methods, which could allow the treatment of subgroups of patients, even in primary care [57]. Another challenge in eliminating HCV infection is the coronavirus (SARS-CoV-2) pandemic. It is estimated that the impact of the pandemic on global efforts to eradicate HCV could lead to an increase in the incidence of almost 45,000 HCC cases [58].

## 3. Primary Liver Cancer

All liver tumors can be divided into benign and malignant. Among the malignant tumors, we can distinguish primary tumors (originating from cells within the liver) and secondary tumors (caused by the appearance of distant metastases from other organs affected by the neoplastic process).

The most frequently occurring primary malignancies of the liver include cancer derived from hepatocytes (hepatocellular carcinoma), and cancer arising from cells that line the bile conductors within the organ. Other malignant tumors in this location are rare. Hepatocellular carcinoma (HCC) and intrahepatic cholangiocarcinoma (cholangiocarcinoma, CCC), although different in the official classifications of the diseases, are classified into one group of ‘primary liver cancer’ [3]. More detailed classification of liver tumors is presented in Table 2.

### 3.1. Epidemiology

Liver cancer is more than twice as likely to affect men than women. The highest incidence of this cancer is observed in the sixth to seventh decade of life. Only in 2020 were there more than 900,000 new cases (approximately 630,000 males and 270,000 females) and more than 800,000 deaths (570,000 males and 250,000 females) for liver cancer worldwide. The distribution of primary liver cancer is characteristic. Most cases are registered in the Far East and subtropical regions of Africa. In Europe, the incidence of primary liver cancer ranges from approximately 5 cases per 100,000 population (Western, Northern and Central Europe) to 10.5 cases per 100,000 population in the Mediterranean region. In recent years, a significant increase in primary liver cancer has been observed in developed countries, especially in Europe and the USA. As GLOBOCAN (Global Cancer Observatory) estimates, primary liver cancer was the sixth most commonly diagnosed cancer and the third leading cause of cancer death worldwide in 2020. Additionally, the highest rates are observed mainly in countries with a lower income, such as Mongolia, Egypt, Thailand, Cambodia and Vietnam [3,59].

### 3.2. Hepatocellular Carcinoma (HCC)

**Figure 1:**
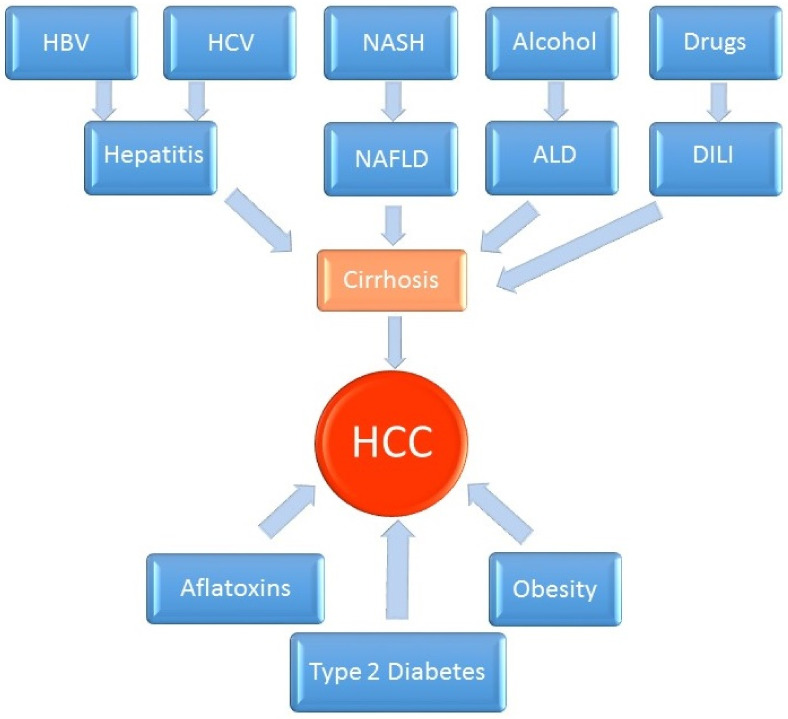
Pathogenesis of hepatocellular carcinoma.

HCC is the most common primary malignancy of the liver. It is derived from hepatocytes, the main mass of the liver parenchyma. This type of liver cancer has the form of a solid tumor that is vascularized by blood from the hepatic artery and is surrounded by a connective tissue capsule. Its most common etiological factor is HBV infection. The development of cirrhosis is associated with a high risk for HCC with most common risk factors including alcohol, HCV infection and NAFLD (Figure 1).

These malignancies represent over 90% of primary liver cancer cases and usually do not show characteristic symptoms. In the majority of hepatocellular carcinoma cases, it develops in patients with cirrhosis [60,61]. The prevalence of HCC increases with age and reaches its peak at around the age of 70. In China and Africa, the average age of onset of HCC is much lower (20–40 years). In contrast, in Japan, the highest incidence of HCC concerns men aged 70–79 years. The highest prevalence of HCC is recorded in eastern Asia, Sub-Saharan Africa and Melanesia (the islands of western Oceania). In the countries of the western hemisphere, the incidence of HCC is lower (with the exception of southern Europe) [59]. The main reason for the increase in the incidence of HCC is the “cohort effect”, originating from the epidemic of hepatitis C infections. Apart from the epidemic of HCV infections, the increase in the incidence of HCC may also be influenced by the hepatitis B virus, mainly in countries with high immigration from endemic regions. In recent years, a decrease in the incidence of HBV has been recorded in endemic countries thanks to the vaccination of children. A further reduction in the incidence of HCC can be expected with the use of orally administered drugs inhibiting the HBV replication activity [62].

Approximately 90% of all HCC cases are associated with known risk factors, as previously described. Interestingly, the risk of HCC development is higher in patients with thrombocytopenia and the presence of esophageal varices, as well as in men and in elderly patients. The risk of HCC is also related to the level of portal pressure and the advancement of liver fibrosis in elastographic measurements. The remaining HCC cases (10%) can be attributed to other etiological factors, which most often also lead to cirrhosis. These include alcoholism, inherited metabolic diseases (e.g., haemochromatosis or alpha-1 antitrypsin deficiency and the metabolic syndrome responsible for non-alcoholic fatty liver disease (NAFLD) [22]. Additionally, HCC occurs sporadically in people with Wilson’s disease and only in the presence of cirrhosis. Exposure to food products contaminated with aflatoxin B1 from *Aspergillus flavus* and *Aspergillus parasitivus* fungi is an important factor in the development of HCC in people living in some regions of Asia and Africa. Epidemiological studies show a strong correlation between HCC and aflatoxin consumption and the presence of abnormal p53 protein (especially in HBV-infected patients) [63,64]. People infected with the human immunodeficiency virus (HIV) have an increased risk of HCC. HIV seems to be an additional risk factor for HCC in patients with chronic hepatitis B and C [65].

### 3.3. Intrahepatic Cholangiocarcinoma (CCC)

The liver produces bile and transmits it via ducts to the gallbladder or directly to the distal part of the digestive tract. Bile ducts are divided into those in the liver and those that are extrahepatic. Cancers of intrahepatic bile ducts together with hepatocellular carcinomas are classified as primary liver cancers. CCC is second most common type of primary liver cancer, which constitute a small percentage of all liver and biliary tract cancers. Intrahepatic cholangiocarcinoma of the small intrahepatic ducts may, for a long time, not show any symptoms, and they are most often detected accidentally during an imaging test (usually ultrasound) performed for other reasons. Large-sized tumors can give non-characteristic symptoms such as aches and pains, followed by pain in the right upper abdomen, lack of appetite, weight loss, weakness, and sometimes increased nocturnal sweating. Depending on the location of the lesion, symptoms of cholestasis may appear with different timing, which depends from the course of the disease. Cholestasis is manifested as pruritus, jaundice and elevated enzyme-labeled liver enzymes [3].

### 3.4. Diagnostics

Histopathological evaluation of focal liver lesions in at-risk groups is recommended for all patients. However, the diagnostic sensitivity of a biopsy depends on the location and size of the focus, the expertise of the pathologist and the skill of the biopsy physician. The morphological features of the cells do not allow early liver cancer to be distinguished from a regenerative nodule with a high degree of dysplasia. Core needle biopsy is more valuable in the diagnosis of this neoplasm. In the tissue material obtained in this way, the architectural assessment, the connective tissue stroma and the presence of small vessel invasions are also assessed. Obtaining representative material during a biopsy allows for immunohistochemical and molecular diagnostics, which is of particular importance in the case of poorly differentiated cancer. Nevertheless, the use of a core needle biopsy carries a risk of bleeding, pain and the possibility of spreading cancer cells along the canal after the biopsy needle [66].

The assessment of liver cancer staging is crucial in selecting the appropriate therapeutic treatment. However, it should be remembered that, despite the use of modern radiological methods for this purpose, the risk of “underestimating” liver cancer advancement is 25–30%. This is because it is prone to vascular infiltration and is often multifocal. Before radical surgical treatment, and especially before liver transplantation, it is recommended to exclude the presence of metastases using chest CT and bone scintigraphy. Unlike most cancers, positron emission tomography (PET) is not an effective diagnostic tool in patients with primary liver cancer. A factor that hinders the characterization of focal lesions is the pathologically “remodeled” liver parenchyma. Interestingly, in the case of HCC, for the first time in the history of clinical oncology, in 2001, in Barcelona, the EASL expert panel established the criteria for the diagnosis of malignant neoplasm solely on the basis of imaging tests and additional examination of serum AFP (alpha fetoprotein) concentration without the need for histological verification [62].

The majority of patients are diagnosed at an advanced stage of the disease, which reduces the chances of survival and cure. Therefore, early detection is extremely important because only patients with non-advanced tumors, without metastases can be treated with the hope of a longer survival. The classical tumor markers—AFP in HCC, or in case of CCC—cancer antigen 19-9 (CA 19-9)—widely recognized as good indices of liver cancers, are not useful for early diagnosis because they neither detect all cases nor are characteristic only for those diseases. Imaging tests such as ultrasound, computed tomography (CT) or magnetic resonance (MR) are considered to be better screening tools than laboratory tests to detect liver cancer. However, the combination of these two methods seems to be the most useful [4]. Therefore, it is very important to search for new markers that would show high diagnostic sensitivity and specificity in the detection of liver cancer in the early stages of the disease. Thus, they might be used as screening tests in primary liver cancer, which would significantly increase the percentage of patients cured.

## 4. Chemokines

Chemokines belong to a group of secreted proteins with the common name of cytokines. They are a large family of about 50 members, which have a low molecular mass (8–12 kDa). Their main role is to direct the recruitment and migration of cells to sites of inflammation or injury [5]. They are divided into four classes (XC, CC, CXC, CX3C) according to the placement and number of cysteine residues at the amino terminus. Each chemokine has a specific receptor located on the cell membrane of target cells (specific effector cells). At present, there are 19 receptors corresponding to specific groups of chemokines [67]. Chemokines and receptor complexes demonstrate widely varying differences in terms of selectivity and binding [68,69]. Some chemokines can bind and activate more than one chemokine receptor and some chemokine receptors can be activated by more than one chemokine ligand [70]. Chemokines are best known for their regulation of leukocyte migration. This is necessary to maintain proper homeostasis of the organism and defense against infiltrating pathogens. Therefore, all chemokines were divided into those related to maintaining homeostasis and chemokines that react during ongoing inflammation [5]. The division of chemokines and their receptors is shown in Table 3.

All chemokines involved in inflammatory reactions may play an important role in the pathogenesis of primary liver cancer. The pathogenesis of cancer in the setting of inflammation and regeneration, as it occurs in chronic liver diseases, anticipates a major role for these proteins and their receptors at different levels [71].

## 5. Chemokines in Liver Neoplasms

As more than 90% of all primary liver tumors are hepatocellular carcinomas (HCCs), this chapter is devoted to the participation of chemokines in the development of this type of cancer. Inflammation is involved in cancer progression. Many factors, such as chemokines, are responsible for the initiation of inflammatory processes influenced by the chemotaxis of monocytes, lymphocytes, neutrophils, eosinophils, basophils, natural killer cells, dendritic cells, and endothelial cells. The second process which can stimulate carcinogenesis is angiogenesis. In this process, various chemokines such as CCL2, CCL5, CXCL8 can be involved. Therefore, all these chemokines can simultaneously contribute to the development of cancer [72]. Until now, the influence of a few chemokines on the initiation in the course of HCC has been discovered. The results of most publications have focused on measurement of several chemokines and their corresponding receptors [73,74,75,76,77]. Some chemokines (CCL2, CCL5, CXCL5, CXCL8) that contribute to the development of inflammation and angiogenesis are supported by other chemokines through participation in fibrosis, HBV/HCV infections, the progression of the tumor and metastasis [73].

Hepatic fibrosis is the consequence of chronic inflammation. Advanced fibrosis leads to liver failure and hepatocellular carcinoma (HCC) [78]. CCL2 and CCR2 are the main promoters of inflammatory monocyte subset accumulation in chronically injured livers. Some studies have confirmed the effects of high concentrations of CCL2 and its receptor on the accumulation of monocytes/macrophages and the initiation of the inflammatory process, as well as the initiation of fibrinogenesis, which clearly contributes to the onset of carcinogenesis [79]. A second important chemokine contributing to the inflammatory, fibrogenic and tumorigenic environment is the chemokine CCL5, which binds to receptors CCR1 and CCR5. Deletion of the CCL5 gene, as well as pharmacological targeting of CCL5 abolishes experimental hepatic fibrosis in mice [73]. Other authors have found that CCL4 and CCL5 levels in the serum of HCC cirrhotic patients were higher than in cases of cirrhotic patients without HCC, which makes them interesting candidates for diagnostic biomarkers of HCC. The performance of CCL4 and CCL5 was comparable for HCC detection with an AUC (area under the curve) for CCL5 of 0.72 and relatively high sensitivity (71%) and specificity (68%) [80].

In the study of Zhu et al. [81], CCL14 expression was analyzed in HCC patients using tissue microarrays. The authors revealed that CCL14 was decreased in tumor tissues compared to peritumoral tissues. Low expression of CCL14 was associated with poor prognosis and was also predictive in liver cirrhosis. The basic mechanisms were further explored by those scientists in HCC cell lines by overexpression of CCL14 and knockdown in vitro. The CCL14 overexpression inhibited proliferation and promoted apoptosis of cancer cells. Finally, the result was confirmed with the use of animal tumor xenograft models in vivo, which resulted in overexpression of CCL14 and led to tumor growth inhibition. These findings suggest that CCL14 could be used as a new prognostic factor for HCC and as a tumor suppressor. Other authors [82] have also determined the expression of CCL14 and its influence on cancer prognosis. These authors showed a significantly lower expression of CCL14 in HCC tissues compared to normal tissues which confirms previous results. Low expression of CCL14 was associated with poor overall survival, disease-specific survival, progression-free survival, and relapse-free survival, especially in the early stages of the disease. These findings show that CCL14 is a potential prognostic biomarker that determines tumor progression in HCC.

Interestingly, in the study of Li et al. [83], the levels of mRNA and CCL15 protein expression in HCC-positive HBV samples were higher than in adjacent liver tissues. In addition, CCL15 was significantly associated with HBx expression and upregulation of HBx-induced CCL15 expression in vitro. High CCL15 expression was significantly associated with a poor prognosis in HCC patients.

Another process leading to liver cancer progression is the recruitment of lymphocytes and tumor-associated macrophage populations by chemokines, which strongly indicates a key role of chemokine pathways in liver carcinogenesis [73]. CCL3, CXCL5, CXCL8 and CXCL12 chemokines are mainly involved in this way. CCL3 interacts with the CCL5/CCR1 or CCL5/CCR5 axes in the inflammation process. Besides this, CCL3 has been identified as recruiting leukocytes to hepatocellular carcinoma. In mice with a deleted CCL3 gene, the number of cases of HCC were remarkably reduced [84]. CXCL5 has been identified as promoting HCC cell proliferation, migration and invasion. These processes took place through the activation of PI3K and ERK1/2 signaling pathways and neutrophil infiltration. Its expression was increased in tumor tissues from patients with HCC when compared to controls, indicating that CXCL5 overexpression has a potential role in therapy of this type of tumor [76]. Some liver macrophages (Kupffer cells) were indicated to secrete the CXCL8 chemokine (via CXCR1 and CXCR2) to neutrophils, which induces hepatocyte necrosis. Some researchers found, that CXCL8 is a potential biomarker of tumor burden and aggressiveness, not only in HCC, but also other types of tumors (i.e., melanoma, renal cell carcinoma and non-small-cell lung cancer [85,86,87]. CXCL12 with its receptor CXCR4 are considered as important factors in the regulation of angiogenesis [88]. High expression of this chemokine and its receptor has been found in HCC patients compared to control groups (cirrhosis, liver adenocarcinoma and normal liver tissues) [89]. Other researchers have suggested that the CXCL12/CXCR4 axis may play an important role in the metastasis of HCC by promoting the migration of tumor cells [90].

In the case of the CXCL14 chemokine, its low expression was found in HCC tissues and cells. The induced overexpression of CXCL14 inhibited cell viability and growth, but promoted apoptosis through the upregulation of Bax and caspase 3, downregulation of Bcl-2, and inhibition of Akt and mTOR phosphorylation. Meanwhile, the knockdown of CXCL14 had the opposite effect to the overexpression of CXCL14, which indicates its low usefulness in therapy [91].

Different authors [92,93,94] performed the mRNA transcriptional and survival analysis of chemokines in HCC patients from different (ONCOMINE, GEPIA, cBioPortal, UALCAN, STRING, GeneMinia, DAVID, Kaplan–Meier plotter, TIMER, GSCALite and NetworkAnalyst) databases. The results of this study showed significantly lower mRNA expression of CXCL1, CXCL2, CXCL5, CXCL6, CXCL7, CXCL12 and CXCL14, and significantly higher mRNA expression levels of CXCL9, CXCL10, CXCL16 and CXCL17 in HCC tissues. Interestingly, survival analysis showed that high levels of CXCL1, CXCL3, CXCL5 and CXCL8 may show poorer overall survival in HCC patients, while high levels of CXCL2 may result in better overall survival. Overall, those studies shows that CXCL1, CXCL2, CXCL5, CXCL6, CXCL7, CXCL9, CXCL10, CXCL12, CXCL14, CXCL16 and CXCL17 may be novel prognostic markers of HCC. On the other hand, CXCL1, CXCL2, CXCL3, CXCL5 and CXCL8 may serve as potential targets for the personalized treatment of HCC.

A similar study was performed in the case of CC chemokines by Jiang et al. [95] where the authors, with the use of ONCOMINE, GEPIA, UALCAN, STRING, WebGestalt, GeneMANIA, TRRUST, DAVID 6.8, LinkedOmics, TIMER, GSCALite, and Open Targets databases, revealed that the transcriptional levels of CCL5, CCL8, CCL11, CCL13, CCL15, CCL18, CCL20, CCL21, CCL25, CCL 26, CCL 27 and CCL28 in HCC tissues were significantly elevated, while CCL2, CCL3, CCL4, CCL14, CCL23 and CCL24 were significantly lowered. Importantly, patients with low levels of CCL14 and CCL21 were associated with a significantly poor prognosis. These results provided potential insight into new targets for immune therapy and prognostic factors for HCC biomarkers.

## 6. Potential Clinical Implications and Future Directions

Due to the poor prognosis for HCC patients, there is an urgent need to identify novel therapeutic strategies. The use of this knowledge seems to be important for the implementation and even greater understanding of the expression mechanisms and role of chemokines for the management of this disease. In recent years, immunotherapy and molecular targeted therapy have become increasingly popular. However, many researchers are also trying to detect parameters that could allow for the early detection of cancer. Interestingly, chemokines and their stimulation of the chemokine receptors expressed in cancer cells have shown them to be regulators of biological processes involved in HCC development. So far, it has been established that some chemokines, including CCL2, CCL4 and CCL5, as well as CCL14, show high diagnostic utility in the detection of HCC [79,80,81]. For this reason, it is also very important to continue research into the usefulness of these proteins and their role in cancer development. It seems particularly important to fully understand the relationships concerning the structure and function of chemokine receptors, the regulation of their signaling pathways, and the genetic and epigenetic mechanisms regulating the expression of chemokines and their receptors. This may create opportunities for the development of new drugs modulating the inflammatory and immune response in the treatment of chronic cancer diseases. Some studies also indicate a significant role of chemokines (including CCL20) in the migration of T_reg_ lymphocytes from neoplastic tissues, which reportedly fosters tumor progression as well as it being associated with poor HCC prognosis. Therefore, comprehensive analyses of cancer tissues in terms of chemokines are necessary for the development of personalized therapy in HCC [96,97]. Some researchers estimate that in the next 5–10 years, the hepato-oncology community will have a broader spectrum of tools to predict HCC, with simple serum assessments [80].

## 7. Conclusions

Hepatocellular carcinoma is the most common primary malignancy of the liver. It is derived from hepatocytes and has the form of a solid tumor that is vascularized by blood from the hepatic artery. Chemokines play a significant role in the process of tumor formation. In HCC, this is mainly carried out by two mechanisms. Inflammation (which leads to organ fibrosis) or recruitment of lymphocytes and tumor-associated macrophage populations. There are many known chemokines, described above, that contribute to the initiation of those processes. Their significant role in the course of HCC proves the high usefulness of those proteins in detection, and in monitoring the course and treatment of HCC.

## Figures and Tables

**Table 1:** The most important risk factors of primary liver cancer.

Risk Factors of Liver Cancer	
Gender	Cirrhosis and conditions that contribute to its initiation
Ethnicity	Non-alcoholic fatty liver disease
Chronic viral hepatitis B and C	Primary biliary cirrhosis
Obesity	Inherited metabolic diseases
Type 2 diabetes	Heavy alcohol use
Aflatoxins	Arsenic
Tobacco use	Infection with parasites
Anabolic steroids Oral contraceptives (the effect is still unclear)	Vinyl chloride and thorium dioxide

**Table 2:** Detailed classification of liver tumors.

Liver Tumors			
Benign			
Hemangioma	Hepatic adenoma		Focal nodular hyperplasia
Tumor			
Primary		Secondary	
Hepatocellular carcinoma		Metastatic liver cancers, mostly pancreatic, colon, stomach, breast or lung cancers	
Intrahepatic cholangiocarcinoma			
Angiosarcoma and hemangiosarcoma			
Hepatoblastoma			

**Table 3:** Chemokines and their receptors.

	Chemokines	Receptors
CC group	CCL1	CCR8
	CCL2, CCL7	CCR2
	CCL3, CCL6, CCL14, CCL9/CCL10, CCL23	CCR1
	CCL4	CCR1, CCR5
	CCL5	CCR5
	CCL8	CCR1, CCR2, CCR5
	CCL11, CCL13	CCR2, CCR3, CCR5
	CCL12, CCL18	Unknown
	CCL15	CCR1, CCR3
	CCL16	CCR1, CCR2, CCR5, CCR8
	CCL17, CCL22	CCR4
	CCL19, CCL21	CCR7
	CCL20	CCR6
	CCL24, CCL26	CCR3
	CCL25	CCR9
	CCL27	CCR10
	CCL28	CCR3, CCR10
CXC group	CXCL1, CXCL2, CXCL3, CXCL5, CXCL7	CXCLR2
	CXCL4, CXCL9, CXCL10	CXCLR3
	CXCL6, CXCL8	CXCLR1, CXCLR2
	CXCL11	CXCLR3, CXCLR7
	CXCL12	CXCLR4, CXCLR7
	CXCL13	CXCLR5
	CXCL14, CXCL15, CXCL17	Unknown
	CXCL16	CXCLR6
CX3C group	CX3CL1	CX3CR1
XC group	XCL1, XCL2	XCR1

## Data Availability

Not applicable.

## References

[1] Juza R.M., Pauli E.M. (2014). Clinical and surgical anatomy of the liver: A review for clinicians. Clin. Anat..

[2] Tajiri K., Shimizu Y. (2013). Liver physiology and liver diseases in the elderly. World J. Gastroenterol..

[3] Wojciechowska U., Didkowska J. Morbidity and Deaths from Malignant Tumors in Poland. Polish National Cancer Registry, Maria Skłodowska—Curie Oncology Centre. http://onkologia.org.pl/raporty/.

[4] Fu J., Wang H. (2018). Precision diagnosis and treatment of liver cancer in China. Cancer Lett..

[5] Legler D.F., Thelen M. (2016). Chemokines: Chemistry, Biochemistry and Biological Function. Chimia.

[6] Rinella M.E. (2015). Nonalcoholic fatty liver disease: A systematic review. JAMA.

[7] Ryu S., Chang Y., Jung H.S., Yun K.E., Kwon M.J., Choi Y., Kim C.W., Cho J., Suh B.S., Cho Y.K. (2015). Relationship of sitting time and physical activity with non-alcoholic fatty liver disease. J. Hepatol..

[8] Estes C., Razavi H., Loomba R., Younossi Z., Sanyal A.J. (2018). Modeling the epidemic of nonalcoholic fatty liver disease demonstrates an exponential increase in burden of disease. Hepatology.

[9] Anstee Q.M., Targher G., Day C.P. (2013). Progression of NAFLD to diabetes mellitus, cardiovascular disease or cirrhosis. Nat. Rev. Gastroenterol. Hepatol..

[10] Harrison A., Christopher Paul D. (2007). Benefits of lifestyle modification in NAFLD. Gut.

[11] Xia H., Zhu X., Zhang X., Jiang H., Li B., Wang Z., Li D., Jin Y. (2019). Alpha-naphthoflavone attenuates non-alcoholic fatty liver disease in oleic acid-treated HepG2 hepatocytes and in high fat diet-fed mice. Biomed. Pharmacother..

[12] Utzschneider K.M., Kahn S.E. (2006). Review: The role of insulin resistance in nonalcoholicfatty liver disease. J. Clin. Endocrinol. Metab..

[13] Metin B., Kcen B.G., Hakan S. (2013). From fatty liver to fibrosis: A tale of second hit. World J. Gastroenterol..

[14] Chung G.E., Kim D., Kim W., Yim J.Y., Park M.J., Kim Y.J., Yoon J.H., Lee H.S. (2012). Non-alcoholic fatty liver disease across the spectrum of hypothyroidism. J. Hepatol..

[15] Eshraghian A., Jahromi A.H. (2014). Non-alcoholic fatty liver disease and thyroid dysfunction: A systematic review. World J. Gastroenterol..

[16] Kaltenbach T.E.M., Graeter T., Oeztuerk S., Holzner D., Kratzer W., Wabitsch M., Denzer C. (2017). Thyroid dysfunction and hepatic steatosis in over-weight children and adolescents. Pediatric Obes..

[17] Matteoni C.A., Younossi Z.M., Gramlich T., Boparai N., Liu Y.C., McCullough A.J. (1999). Nonalcoholic fatty liver disease: A spectrum of clini-cal and pathological severity. Gastroenterology.

[18] Alisi A., Feldstein A.E., Villani A., Raponi M., Nobili V. (2012). Pediatric non-alcoholic fatty liver disease: A multidisciplinary approach. Nat. Rev. Gastroenterol. Hepatol..

[19] Pacifico L., Nobili V., Anania C., Verdecchia P., Chiesa C. (2011). Pediatric non-alcoholic fatty liver disease, metabolic syndrome and cardiovascularrisk. World J. Gastroenterol..

[20] Kanwal F., Kramer J.R., Mapakshi S., Natarajan Y., Chayanupatkul M., Richardson P.A., Li L., Desiderio R., Thrift A.P., Asch S.M. (2018). Risk of Hepatocellular Cancer in Patients With Non-Alcoholic Fatty Liver Disease. Gastroenterology.

[21] Gudowska M., Wojtowicz E., Cylwik B., Gruszewska E., Chrostek L. (2015). The Distribution of Liver Steatosis, Fibrosis, Steatohepatitis and Inflammation Activity in Alcoholics According to FibroMax Test. Adv. Clin. Exp. Med..

[22] Chrostek L., Przekop D., Gruszewska E., Gudowska-Sawczuk M., Cylwik B. (2019). Noninvasive Indirect Markers of Liver Fibrosis in Alcoholics. BioMed Res. Int..

[23] Stickel F., Datz C., Hampe J., Bataller R. (2017). Pathophysiology and management of alcoholic liver disease: Update 2016. Gut Liver.

[24] Dunn W., Shah V.H. (2016). Pathogenesis of alcoholic liver disease. Clin. Liver Dis..

[25] Gao B., Bataller R. (2011). Alcoholic liver disease: Pathogenesis and new therapeutic targets. Gastroenterology.

[26] Kourkoumpetis T., Sood G. (2019). Pathogenesis of Alcoholic Liver Disease: An Update. Clin. Liver Dis..

[27] Farooq M.O., Bataller R. (2016). Pathogenesis and Management of Alcoholic Liver Disease. Dig. Dis..

[28] Sheron N. (2016). Alcohol and liver disease in Europe–Simple measures have the potential to prevent tens of thousands of premature deaths. J. Hepatol..

[29] Dunn W., Angulo P., Sanderson S., Jamil L.H., Stadheim L., Rosen C., Malinchoc M., Kamath P.S., Shah V.H. (2006). Utility of a new model to diagnose an alcohol basis for steatohepatitis. Gastroenterology.

[30] Kong L.Z., Chandimali N., Han Y.H., Lee D.H., Kim J.S., Kim S.U., Kim T.D., Jeong D.K., Sun H.N., Lee D.S. (2019). Pathogenesis, Early Diagnosis, and Therapeutic Management of Alcoholic Liver Disease. Int. J. Mol. Sci..

[31] Osna N.A., Donohue T.M., Kharbanda K.K. (2017). Alcoholic Liver Disease: Pathogenesis and Current Management. Alcohol Res. Curr. Rev..

[32] Kullak-Ublick G.A., Andrade R.J., Merz M., End P., Benesic A., Gerbes A.L., Aithal G.P. (2017). Drug-induced liver injury: Recent advances in diagnosis and risk assessment. Gut.

[33] Robles-Díaz M., Medina-Caliz I., Stephens C., Andrade R.J., Lucena M.I. (2016). Biomarkers in DILI: One More Step Forward. Front. Pharmacol..

[34] Thakkar S., Chen M., Fang H., Liu Z., Roberts R., Tong W. (2018). The Liver Toxicity Knowledge Base (LKTB) and drug-induced liver injury (DILI) classification for assessment of human liver injury. Expert Rev. Gastroenterol. Hepatol..

[35] Antoine D.J., Dear J.W., Lewis P.S., Platt V., Coyle J., Masson M., Thanacoody R.H., Gray A.J., Webb D.J., Moggs J.G. (2013). Mechanistic biomarkers provide early and sensitive detection of acetaminophen-induced acute liver injury at first presentation to hospital. Hepatology.

[36] Antoine D.J., Jenkins R.E., Dear J.W., Williams D.P., McGill M.R., Sharpe M.R., Craig D.G., Simpson K.J., Jaeschke H., Park B.K. (2012). Molecular forms of HMGB1 and keratin-18 as mechanistic biomarkers for mode of cell death and prognosis during clinical acetaminophen hepatotoxicity. J. Hepatol..

[37] Antoine D.J., Williams D.P., Kipar A., Jenkins R.E., Regan S.L., Sathish J.G., Kitteringham N.R., Park B.K. (2009). High-mobility group box-1 protein and keratin-18, circulating serum proteins informative of acetaminophen-induced necrosis and apoptosis in vivo. Toxicol. Sci..

[38] Schomaker S., Warner R., Bock J., Johnson K., Potter D., Van Winkle J., Aubrecht J. (2013). Assessment of emerging biomarkers of liver injury in human subjects. Toxicol. Sci..

[39] Harrill A.H., Roach J., Fier I., Eaddy J.S., Krutz C.L., Antoine D.J., Spencer D.M., Kishimoto T.K., Pisetsky D.S., Park B.K. (2012). The effects of heparins on the liver: Application of mechanistic serum biomarkers in a randomized study in healthy volunteers. Clin. Pharmacol. Ther..

[40] Thulin P., Nordahl G., Gry M., Yimer G., Aklillu E., Makonnen E., Aderaye G., Lindquist L., Mattsson C.M., Ekblom B. (2014). Keratin-18 and microRNA-122 complement alanine aminotransferase as novel safety biomarkers for drug-induced liver injury in two human cohorts. Liver Int..

[41] Wang J.B., Pu S.B., Sun Y., Li Z.F., Niu M., Yan X.Z., Zhao Y.L., Wang L.F., Qin X.M., Ma Z.J. (2014). Metabolomic profiling of autoimmune hepatitis: The diagnostic utility of nuclear magnetic resonance spectroscopy. J. Proteome Res..

[42] Ward J., Kanchagar C., Veksler-Lublinsky I., Lee R.C., McGill M.R., Jaeschke H., Curry S.C., Ambros V.R. (2014). Circulating microRNA profiles in human patients with acetaminophen hepatotoxicity or ischemic hepatitis. Proc. Natl. Acad. Sci. USA.

[43] Starkey Lewis P.J., Dear J., Platt V., Simpson K.J., Craig D.G., Antoine D.J., French N.S., Dhaun N., Webb D.J., Costello E.M. (2011). Circulating microRNAs as potential markers of human drug-induced liver injury. Hepatology.

[44] Onji M., Fujioka S., Takeuchi Y., Takaki T., Osawa T., Yamamoto K., Itoshima T. (2009). Clinical characteristics of drug-induced liver injury in the elderly. Hepatol. Res..

[45] Aggarwal R., Goel A. (2015). Hepatitis A: Epidemiology in resource-poor countries. Curr. Opin. Infect. Dis..

[46] Thuener J. (2017). Hepatitis A and B Infections. Fam. Med. Prim. Care.

[47] Yan Z., Wang Y. (2017). Viral and host factors associated with outcomes of hepatitis C virus infection (Review). Mol. Med. Rep..

[48] Wang L.S., D’Souza L.S., Jacobson I.M. (2016). Hepatitis C-A clinical review. J. Med. Virol..

[49] Guerra J.A.A.A., Kampa K.C., Morsoletto D.G.B., Junior A.P., Ivantes C. (2017). Hepatitis E: A Literature Review. J. Clin. Transl. Hepatol..

[50] European Association for the Study of the Liver (2018). EASL Clinical Practice Guidelines: Management of hepatocellular carcinoma. J. Hepatol..

[51] Gudowska M., Gruszewska E., Panasiuk A., Cylwik B., Swiderska M., Filisiak R., Szmitkowski M., Chrostek L. (2017). Changed Profile of Serum Transferrin Isoforms in Liver Diseases. Clin. Lab..

[52] Chang M.H. (2014). Prevention of hepatitis B virus infection and liver cancer. Recent Results in Cancer Res..

[53] Kanwal F., Kramer J.R., Ilyas J., Duan Z., El-Serag H.B. (2014). HCV genotype 3 is associated with an increased risk of cirrhosis and hepatocellular cancer in a national sample of U.S. Veterans with HCV. Hepatology.

[54] De Mattos Â.Z., Debes J.D., Boonstra A., Yang J.D., Balderramo D.C., Sartori G.D.P., de Mattos A.A. (2021). Current impact of viral hepatitis on liver cancer development: The challenge remains. World J. Gastroenterol..

[55] Goossens N., Negro F. (2014). Is genotype 3 of the hepatitis C virus the new villain?. Hepatology.

[56] Centre for Disease Analysis (2018). WHO Estimates of the Prevalence and Incidence of Hepatitis C Virus Infection by World Health Organization Region, 2015.

[57] Pawlotsky J.M., Ramers C.B., Dillon J.F., Feld J.J., Lazarus J.V. (2020). Simplification of Care for Chronic Hepatitis C Virus Infection. Semin. Liver Dis..

[58] Blach S., Kondili L.A., Aghemo A., Cai Z., Dugan E., Estes C., Gamkrelidze I., Ma S., Pawlotsky J.M., Razavi-Shearer D. (2021). Impact of COVID-19 on global HCV elimination efforts. J. Hepatol..

[59] Sung H., Ferlay J., Siegel R.L., Laversanne M., Soerjomataram I., Jemal A., Bray F. (2021). Global Cancer Statistics 2020: GLOBOCAN Estimates of Incidence and Mortality Worldwide for 36 Cancers in 185 Countries. CA A Cancer J. Clin..

[60] Shiani A., Narayanan S., Pena L., Friedman M. (2017). The Role of Diagnosis and Treatment of Underlying Liver Disease for the Prognosis of Primary Liver Cancer. Cancer Control..

[61] Ghouri Y.A., Mian I., Rowe J.H. (2017). Review of hepatocellular carcinoma: Epidemiology, etiology, and carcinogenesis. J. Carcinog..

[62] Łapiński T.W., Tarasik A., Januszkiewicz M., Flisiak R. (2021). Clinical aspects and treatment of hepatocellular carcinoma in north-eastern Poland. Clin. Exp. Hepatol..

[63] Polio J., Enriquez R.E., Chow A., Wood W.M., Atterbury C.E. (1989). Hepatocellular carcinoma in Wilson’s disease. Case report and review of the literature. J. Clin. Gastroenterol..

[64] Hsu I.C., Metcalf R.A., Sun T., Welsh J.A., Wang N.J., Harris C.C. (1991). Mutational hotspot in the p53 gene in human hepatocellular carcinomas. Nature.

[65] Marcellin P., Pequignot F., Delarocque-Astagneau E., Zarski J.P., Ganne N., Hillon P., Antona D., Bovet M., Mechain M., Asselah T. (2008). Mortality related to chronic hepatitis B and chronic hepatitis C in France: Evidence for the role of HIV coinfection and alcohol consumption. J. Hepatol..

[66] Sergi C.M. (2021). Liver Cancer.

[67] Ridiandries A., Tan J.T., Bursill C.A. (2016). The Role of CC-Chemokines in the Regulation of Angiogenesis. Int. J. Mol. Sci..

[68] Nomenclature IWSoC (2003). Chemokine/chemokine receptor nomenclature. Cytokine.

[69] Schall T.J., Proudfoot A.E. (2011). Overcoming hurdles in developing successful drugs targeting chemokine receptors. Nat. Rev. Immunol..

[70] Vinader V., Afarinkia K. (2012). A beginner’s guide to chemokines. Future Med. Chem..

[71] Marra F., Tacke F. (2014). Roles for chemokines in liver disease. Gastroenterology.

[72] Proost P., Wuyts A., van Damme J. (1996). The role of chemokines in inflammation. Int. J. Clin. Lab. Res..

[73] Ehling J., Tacke F. (2016). Role of chemokine pathways in hepatobiliary cancer. Cancer Lett..

[74] Shih Y.T., Wang M.C., Zhou J., Peng H.H., Lee D.Y., Chiu J.J. (2015). Endothelial progenitors promote hepatocarcinoma intrahepatic metastasis through monocyte chemotactic protein-1 induction of microRNA-21. Gut.

[75] Hou K.Z., Fu Z.Q., Gong H. (2015). Chemokine ligand 20 enhances progression of hepatocellular carcinoma via epithelial-mesenchymal transition. World J. Gastroenterol..

[76] García-Irigoyen O., Latasa M.U., Carotti S., Uriarte I., Elizalde M., Urtasun R., Vespasiani-Gentilucci U., Morini S., Benito P., Ladero J.M. (2015). Matrix metalloproteinase 10 contributes to hepatocarcinogenesis in a novel crosstalk with the stromal derived factor 1/C-X-C chemokine receptor 4 axis. Hepatology.

[77] Bishayee A. (2014). The role of inflammation and liver cancer. Adv. Exp. Med. Biol..

[78] Kocabayoglu P., Friedman S.L. (2013). Cellular basis of hepatic fibrosis and its role in inflammation and cancer. Front. Biosci..

[79] Tacke F. (2012). Functional role of intrahepatic monocyte subsets for the progression of liver inflammation and liver fibrosis in vivo. Fibrogenesis Tissue Repair.

[80] Debes J.D., Romagnoli P.A., Prieto J., Arrese M., Mattos A.Z., Boonstra A. (2021). On Behalf Of The Escalon Consortium. Serum Biomarkers for the Prediction of Hepatocellular Carcinoma. Cancers.

[81] Zhu M., Xu W., Wei C., Huang J., Xu J., Zhang Y., Zhao Y., Chen J., Dong S., Liu B. (2019). CCL14 serves as a novel prognostic factor and tumor suppressor of HCC by modulating cell cycle and promoting apoptosis. Cell Death Dis..

[82] Gu Y., Li X., Bi Y., Zheng Y., Wang J., Li X., Huang Z., Chen L., Huang Y., Huang Y. (2020). CCL14 is a prognostic biomarker and correlates with immune infiltrates in hepatocellular carcinoma. Aging.

[83] Li Y., Wang C., Zhao T., Cui R., Hu L., Chang L., Wei X., Zhang J., Li Y. (2021). Hepatitis B Virus X Protein Modulates Chemokine CCL15 Upregulation in Hepatocellular Carcinoma. Anti Cancer Agents Med. Chem..

[84] Yang X., Lu P., Fujii C., Nakamoto Y., Gao J.L., Kaneko S., Murphy P.M., Mukaida N. (2006). Essential contribution of a chemokine, CCL3, and its receptor, CCR1, to hepatocellular carcinoma progression. Int. J. Cancer.

[85] Ren Y., Poon R.T., Tsui H.T., Chen W.H., Li Z., Lau C., Yu W.C., Fan S.T. (2003). Interleukin-8 serum levels in patients with hepatocellular carcinoma: Correlations with clinicopathological features and prognosis. Clin. Cancer Res..

[86] Sanmamed M.F., Carranza-Rua O., Alfaro C., Oñate C., Martín-Algarra S., Perez G., Landazuri S.F., Gonzalez A., Gross S., Rodriguez I. (2014). Serum interleukin-8 reflects tumor burden and treatment response across malignancies of multiple tissue origins. Clin. Cancer Res..

[87] Zimmermann H.W., Tacke F. (2011). Modification of chemokine pathways and immune cell infiltration as a novel therapeutic approach in liver inflammation and fibrosis. Inflamm. Allergy Drug Targets.

[88] Huang F., Geng X.P. (2010). Chemokines and hepatocellular carcinoma. World J. Gastroenterol..

[89] Neve Polimeno M., Ierano C., D’Alterio C., Simona Losito N., Napolitano M., Portella L., Scognamiglio G., Tatangelo F., Maria Trotta A., Curley S. (2015). CXCR4 expression affects overall survival of HCC patients whereas CXCR7 expression does not. Cell. Mol. Immunol..

[90] Liu H., Pan Z., Li A., Fu S., Lei Y., Sun H., Wu M., Zhou W. (2008). Roles of chemokine receptor 4 (CXCR4) and chemokine ligand 12 (CXCL12) in metastasis of hepatocellular carcinoma cells. Cell. Mol. Immunol..

[91] Bi J., Liu Q., Sun Y., Hu X., He X., Xu C. (2021). CXCL14 inhibits the growth and promotes apoptosis of hepatocellular carcinoma cells via suppressing Akt/mTOR pathway. J. Recept. Signal Transduct..

[92] Wang Y.H., Huang J.H., Tian Z.F., Zhou Y.F., Yang J. (2019). The role of CXC cytokines as biomarkers and potential targets in hepatocellular carcinoma. Math. Biosci. Eng..

[93] Wang J., Zhang C., Chen X., Li Y., Li A., Liu D., Li F., Luo T. (2021). Functions of CXC chemokines as biomarkers and potential therapeutic targets in the hepatocellular carcinoma microenvironment. Transl. Cancer Res..

[94] Lin T., Zhang E., Mai P.P., Zhang Y.Z., Chen X., Peng L.S. (2021). CXCL2/10/12/14 are prognostic biomarkers and correlated with immune infiltration in hepatocellular carcinoma. Biosci Rep..

[95] Jiang Z., Xing C., Wang P., Liu X., Zhong L. (2021). Identification of Therapeutic Targets and Prognostic Biomarkers Among Chemokine (C-C Motif) Ligands in the Liver Hepatocellular Carcinoma Microenvironment. Front. Cell Dev. Biol..

[96] Xue D., Zheng Y., Wen J., Han J., Tuo H., Liu Y., Peng Y. (2021). Role of chemokines in hepatocellular carcinoma (Review). Antioxid. Redox Signal..

[97] Nishida N., Kudo M. (2017). Immunological Microenvironment of Hepatocellular Carcinoma and Its Clinical Implication. Oncology.

